# Carbon Isotope Ratios as Biomarkers for Added Sugar Intake: Fundamental Limitations that Constrain Valid Application

**DOI:** 10.1016/j.tjnut.2026.101575

**Published:** 2026-05-04

**Authors:** Jimmy Chun Yu Louie

**Affiliations:** Discipline of Dietetics, Department of Allied Health, School of Health Sciences, Swinburne University of Technology, Hawthorn, Victoria, Australia

**Keywords:** carbon isotope ratio, added sugars, free sugars, biomarkers, validity

## Abstract

Carbon isotope ratio (CIR; δ^13^C) analysis has been proposed as a biomarker for added sugar intake from C4 sources, but its specificity is limited. CIR biomarkers measure total C4 dietary carbon, conflating added sugars with other C4 foods like corn-fed meats and whole corn products. This lack of specificity varies across populations and food systems. Moreover, metabolic redistribution of dietary carbon means that δ^13^C signals in tissues reflect complex mixtures of sources and processes, not sugar intake alone. Attempts to adjust for confounders rely on self-reported dietary data, undermining the objectivity that CIR biomarkers aim to provide. Even under ideal conditions, CIR validity is insufficient for most nutritional research, especially when assessing causal relationships or meaningful effects. The shift in public health focus from “added sugars” to “free sugars,” including C3-derived sources like fruit juice and honey, further limits CIR utility. Although CIR has proven useful for distinguishing dietary protein sources (e.g., marine compared with terrestrial), its application to added sugars faces persistent challenges. Resources may be better directed toward improving dietary assessment tools or developing more specific metabolomic markers, acknowledging that all current methods have limitations.

## Introduction

The well-documented inadequacies of self-reported dietary assessment have motivated an extensive search for objective biomarkers, particularly for socially undesirable foods like added sugars [1,2]. Carbon isotope ratio (CIR; δ^13^C) analysis emerged as an appealing solution by exploiting fundamental differences in plant photosynthetic pathways. C4 plants (including corn and sugarcane) use a carbon-concentrating mechanism that preferentially incorporates the heavier ^13^C isotope, resulting in δ^13^C values of approximately –12‰, whereas C3 plants (including most fruits, vegetables, wheat, and beets) discriminate more strongly against ^13^C, yielding δ^13^C values of approximately –27‰ [3,4]. This 15‰ difference is preserved in dietary carbon and incorporated into body tissues, with tissue δ^13^C values reflecting the proportion of C4 compared with C3 carbon in the diet. Because corn and sugarcane dominate added sugar sources in industrialized diets while most other dietary components derive from C3 plants, this isotopic difference appeared large enough to provide a robust signal despite biological noise, leading to substantial research investment in CIR added sugar biomarker development and validation.

CIR biomarkers rely on isotope ratio mass spectrometry (IRMS) to measure the relative abundance of ^13^C to ^12^C in biological specimens, expressed as δ^13^C values in parts per thousand (‰) relative to an international standard [5]. Common specimen types include serum or plasma amino acids (particularly alanine), red blood cells, hair, fingernails, and exhaled breath CO_2_ [6]. Recent systematic reviews have identified δ^13^C-alanine as the most promising CIR biomarker for added sugar intake [6,7], with reported correlations ranging from *R*^2^ = 0.36 to 0.91, depending on study design and population [6]. Pooled analyses across diverse populations demonstrate moderate associations between CIR-alanine and both added sugar (*r* = 0.54) and sugar-sweetened beverage (SSB) (*r* = 0.63) intake [8], whereas controlled feeding studies show that breath CIR responds to both short-term and long-term changes in added sugar and animal protein consumption [9]. Indeed, carbon isotope approaches have proven valuable for assessing dietary components with clear source separation, such as distinguishing marine compared with terrestrial protein sources or quantifying animal compared with plant protein intake, where isotopic signatures map directly onto the dietary exposures of interest [10,11].

**FIGURE 1:**
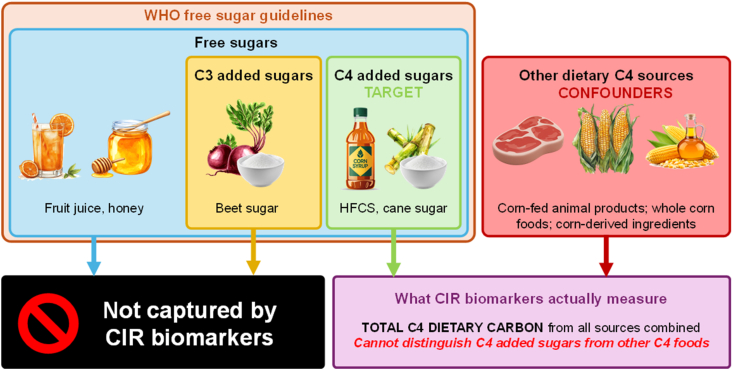
Limitations of CIR biomarkers in identifying C4 added sugars. Images used in this figure were reproduced with permission from Adobe Stock Images (https://stock.adobe.com/au/) under the Education License held by the author’s institution. CIR, carbon isotope ratio; HFCS, high-fructose corn syrup.

Although these recent findings demonstrate detectable associations between CIR biomarkers and added sugar intake under controlled conditions, the fundamental premise underlying CIR biomarkers for added sugar assessment is problematic in ways that limit their utility for most research applications (Table 1). These biomarkers measure total C4 dietary carbon exposure, which includes added sugars but also whole corn foods, corn-fed animal products, corn-derived oils and starches, and other C4 plant materials (Figure 1) [12,13]. The correlation between CIR biomarkers and reported added sugar intake observed in validation studies—although statistically significant—does not necessarily reflect biomarker specificity for added sugars but may instead reflect the demographic fact that in selected study populations consuming minimal whole corn, added sugars happen to be the dominant C4 source, a contingent property of specific food systems rather than an intrinsic property of the biomarker itself. Notably, even systematic reviews concluding that CIR-alanine represents the “most promising” available biomarker acknowledge that all studies to date have been conducted in United States populations, limiting generalizability to regions with different food systems and agricultural practices [6,7].

**TABLE 1:** Fundamental limitations of carbon isotope ratio biomarkers for added sugars

Problem category	Specific issues	Why it is fundamental	Why could not it be fixed?
Dietary specificity	Cannot distinguish added sugars from whole corn foods or corn-based ingredients	Both are C4 plants; isotopes measure photosynthetic pathway, not food processing status	Plant biochemistry is immutable; C4 carbon is C4 carbon regardless of whether it comes from HFCS or tortillas
	Geographic variation in sugar sources (45% beet sugar in some regions)	Beet sugar is C3-derived and isotopically identical to other C3 foods	Would require perfect knowledge of regional food supply and individual sourcing, which defeats the purpose of objective measurement
	Corn-fed animal products contribute C4 signal	Industrial livestock consume corn-based feed; their tissues carry C4 signature independent of consumers’ sugar intake	Would require detailed knowledge of animal feeding practices for every food consumed, which is impossible in practice
Metabolic redistribution	Carbon from all dietary sources mixed through citric acid cycle	Once in central metabolism, carbon atoms from sugars become indistinguishable from carbon from starches, proteins, or fats	This is fundamental biochemistry; metabolic pathways cannot preserve source identity through oxidative and biosynthetic processes
	Gluconeogenesis adds C3 carbon to glucose-derived metabolites	During fasting/exercise, amino acids and glycerol contribute to glucose pool with C3 signature	Normal metabolic regulation; cannot be prevented without disrupting physiology
	Variable incorporation across individuals	Genetic variation, metabolic phenotype, nutritional state affects carbon routing	Human metabolic diversity is fundamental; cannot be eliminated through better measurement
Validation circularity	Reference standard (self-report) has the same errors biomarker aims to avoid	Validating against imperfect self-report cannot establish true validity	This is a logical problem; no amount of additional validation against self-report can break circularity
	Adjustment for confounders requires measuring them via self-report	Must assess corn intake, animal products via dietary recall to “correct” biomarker	If we had good-quality dietary data for confounders, we would not need expensive biomarkers
	Population-specific validity requires continuous revalidation	Food systems, agricultural practices, dietary patterns vary by population and time	This limitation reflects real-world dietary complexity; cannot be overcome

Abbreviation: HFCS, high-fructose corn syrup.

In addition, current public health guidelines have shifted focus from “added sugars” to “free sugars,” a broader category defined by the WHO that includes sugars added to foods plus sugars naturally present in honey, syrups, fruit juices, and fruit juice concentrates [12,14]. Because fruit juices and honey typically derive from C3 plants in most markets [[15], [16], [17]], CIR added sugar biomarkers would systematically underestimate free sugar intake, diminishing their utility for public health surveillance aligned with contemporary WHO recommendations [12,13].

The metabolic fate of dietary carbon further undermines specificity [12,13]. Once consumed, dietary carbohydrates are broken down and their carbon atoms redistributed throughout metabolism. The carbon isotope composition of any biological sample therefore reflects the aggregate isotopic signature of all dietary macronutrients after extensive metabolic processing, not a specific record of added sugar consumption [18].

This review evaluates whether CIR biomarkers provide sufficient validity for common research applications, considering both their theoretical limitations and empirical performance. It is acknowledged that “sufficient validity” is context-dependent and differs by research objective. Therefore, the CIR added sugar biomarker was evaluated against validity requirements for several common applications: population surveillance, etiologic research on sugar–disease associations, and dietary intervention trials. The central argument is that in most research contexts, the specificity and metabolic limitations of CIR added sugar biomarker constrain their validity below levels needed for meaningful inference, and that the required adjustments for confounding create circular dependence on self-report that negates the primary advantage of objective biomarkers. Although the focus was on limitations, it is acknowledged that niche applications may exist where CIR biomarkers provide value, and conditions that might favor their use were discussed.

### The specificity problem: confounding by other C4 sources

#### Geographic variation in sugar sources

The interpretive logic of CIR added sugar biomarkers rests on the assumption that measuring C4 dietary exposure is equivalent to measuring added sugar intake. This assumption is immediately problematic because the United States sugar supply consists of ∼55% C4 sources [cane sugar, high-fructose corn syrup (HFCS)] and 45% C3 beet sugar, with substantial regional and temporal variation [19,20]. European countries derive 30% to 60% of sugar from beets [21]. This means CIR biomarkers systematically miss 45% to 55% of added sugar intake in many populations, a specificity problem that fundamentally limits their utility before considering any other issues [12].

Although this may be corrected by documenting regional sugar sourcing patterns, such corrections require detailed knowledge of: *1*) the geographic distribution of food purchases, *2*) the sugar sources used by food manufacturers whose products are consumed in each region, and *3*) individual-level variation in consumption of products with different sugar sources. The first 2 require extensive food industry and market research data, whereas the third requires either dietary recalls (self-report) or detailed food purchase records. At this point, the CIR added sugar biomarkers provide information contingent on extensive external data collection, reducing their value as an independent measurement.

More fundamentally, the C4/C3 sugar ratio has changed dramatically over time as HFCS displaced cane and beet sugars beginning in the 1970s [[22], [23], [24]], and continues to vary with commodity prices, trade policies, and manufacturing decisions. Between 1970 and 2000, the proportion of sweeteners from HFCS increased from <1% to ∼40% in the United States [24]. This temporal instability means that CIR biomarkers measured at different time periods reflect not only dietary behavior but also agricultural market dynamics. A biomarker whose interpretation depends on current food industry practices and policy environments has limited utility for historical comparisons or prospective studies spanning decades.

Individual-level variation in sugar source exposure compounds these problems. Consumers who preferentially purchase imported foods, organic products, or products from specific manufacturers may have substantially different C4/C3 sugar ratios than population mean suggest. Without measuring individual food sourcing patterns, which requires a detailed dietary assessment, researchers cannot determine whether an individual’s CIR biomarker value reflects high consumption of C4 sugars or preferential consumption of C3-sugar-containing products.

#### Whole corn consumption

Whole corn consumption creates insurmountable confounding in vast populations where maize is a dietary staple, including hundreds of millions of people across Latin America, sub-Saharan Africa, and Indigenous communities worldwide [25]. In these contexts, CIR biomarkers likely provide essentially no information about added sugar intake, instead primarily reflecting the extent to which traditional corn-based foodways persist. Defenders of CIR added sugar biomarkers acknowledge this limitation but argue that the biomarkers remain valid in populations with low whole corn consumption, primarily industrialized Western populations [3,6,9,26]. However, this defense concedes that the biomarker lacks universal applicability and requires a priori knowledge about dietary patterns, the very knowledge that dietary assessment methods aim to provide. Moreover, even in industrialized populations, the assumption of minimal whole corn consumption requires continuous validation. Corn tortillas, polenta, grits, corn cereals, popcorn, and corn-based snack foods contribute a C4 signal that varies by ethnicity, region, socioeconomic status, and food trends [27]. Validation studies conducted in 1 population at 1 time point cannot guarantee that whole corn consumption remains negligible in other populations or future time periods.

The standard approach of adjusting for self-reported total corn intake simply replaces one self-report problem (added sugar intake) with another (whole corn food intake). If researchers possess accurate self-reported data for corn foods, they likely also possess reasonably accurate data for added sugars, given that both are measured through the same dietary assessment instruments. An alternative approach uses the CIR of multiple amino acids to distinguish added sugar from animal protein intake: the CIR of alanine (a nonessential amino acid synthesized from glucose) reflects primarily added sugar intake, whereas the CIR of essential amino acids (which must derive from dietary protein) reflects meat intake [28]. Although this amino acid approach demonstrates improved specificity by showing no association between biomarker-estimated added sugar and meat intake, it still requires self-reported data on participant characteristics (body weight, physical activity, smoking status) to achieve adequate validity (*R*^2^ = 0.37). The biomarker then provides marginal information beyond what is already available through self-report, while adding substantial cost and complexity.

#### Industrial animal agriculture

Modern confined feeding operations rely heavily on corn-based feeds, causing meat, dairy, and eggs from these systems to carry strong C4 isotopic signatures [4,29]. The contribution of animal products to individual CIR biomarkers depends on: *1*) the quantity of animal products consumed, *2*) whether animals were grain-fed compared with pasture-raised, *3*) the proportion of corn compared with other grains in feed, and *4*) tissue-specific routing of dietary carbon in different animal species. Each of these variables exhibits substantial interindividual variation and requires measurement through dietary recall or food sourcing data.

Access to pasture-raised compared with grain-raised animal products correlates with socioeconomic status in many populations [30], meaning CIR biomarkers potentially conflate added sugar intake with economic access to premium foods. Higher-income individuals consuming grass-fed beef and pastured poultry may show lower δ^13^C values independent of added sugar intake, whereas lower-income individuals consuming conventional animal products may show elevated values independent of sugar consumption. Without comprehensive dietary data on animal product consumption and sourcing, researchers cannot disentangle these pathways.

Studies that adjust for self-reported animal product intake face the same circularity problem as adjustments for corn intake: if self-report is sufficiently accurate to measure animal products for adjustment, it is likely accurate enough to directly measure added sugars, rendering the expensive biomarker measurement redundant. Moreover, measurement error in self-reported animal intake, which is substantial [2], produces residual confounding that biases the apparent sugar–biomarker association in unpredictable ways [31,32].

#### Corn-derived food ingredients

Beyond whole corn and animal products, modern food processing introduces numerous corn-derived ingredients that contribute C4 carbon: corn oil, corn starch, corn syrup solids, maltodextrin from corn, dextrose from corn, modified food starch often from corn, and numerous other corn-based additives [33]. These ingredients appear across diverse food categories, including salad dressings, baked goods, processed meats, sauces, and convenience foods [34]. Their contribution to individual CIR biomarkers varies with overall consumption of processed foods, manufacturer ingredient choices (which change over time), and individual food selection patterns. Measuring these diverse corn-derived ingredients requires either a comprehensive dietary recall with detailed ingredient-level coding or extensive food purchase data with complete ingredient databases [31,32]. Both approaches require substantial resources and ultimately depend on dietary data collection. The CIR added sugar biomarkers thus become a complex composite measure whose interpretation requires the very dietary information it was meant to provide independently.

### Free sugars and public health relevance

The WHO definition of “free sugars” includes not only added sugars but also sugars naturally present in honey, syrups, and fruit juices [12,14]. This broader category better captures the sugars associated with adverse health outcomes and forms the basis for current dietary recommendations (consume <10% of energy from free sugars) [14,35,36]. Honey typically derives from the nectar of C3 plants in major production regions [15], and the commonly consumed fruit juices (e.g., apple, orange, grape) come from C3 plants [16]. A substantial proportion of free sugar intake, therefore, has C3 isotopic signatures that CIR biomarkers cannot detect.

The proportion of free sugars that are C3-derived varies by population dietary patterns but likely ranges from 20% to 40% in industrialized countries where fruit juice consumption is common [17]. In populations following dietary guidance to reduce added sugars by substituting fruit juice or honey, CIR biomarkers would systematically underestimate free sugar intake [12,13], potentially leading to erroneous conclusions about compliance with recommendations or associations between free sugar intake and health outcomes. This limitation applies regardless of how successfully other specificity problems are addressed.

### Metabolic redistribution

**FIGURE 2:**
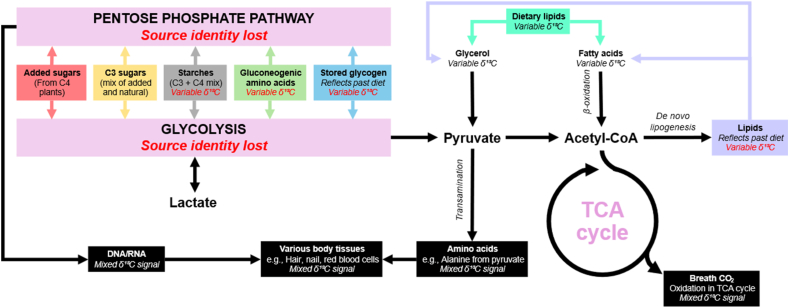
Metabolic fates of dietary nutrients and their Carbon isotope ratio (δ^13^C) contributions to δ^13^C signals in tissues and breath CO_2_. TCA, tricarboxylic acid.

Extensive metabolic redistribution of dietary carbon fundamentally raises theoretical concerns about whether carbon isotopic measurements can track added sugar intake specifically. Once dietary carbohydrates enter metabolism, metabolic pathways have the potential to mix and redistribute carbon atoms from different sources, which would complicate source tracking (Figure 2) [37].

Glucose from added sugars, starches, and gluconeogenesis all enter glycolysis to form pyruvate, which can be: *1*) oxidized via the citric acid cycle to CO_2_, *2*) converted to lactate, *3*) transaminated to form amino acids, and *4*) used as substrate for glycogenolysis and de novo lipogenesis. Theoretical considerations suggest that the citric acid cycle is particularly problematic for source tracking because its cyclic nature and multiple entry points create thorough mixing of carbon from carbohydrates, fatty acids, and amino acids before incorporation into biosynthetic precursors [38]. However, the extent to which this metabolic mixing obscures dietary signals compared with allowing their detection remains an empirical question yet to be addressed by validation studies.

This metabolic architecture means that the isotopic composition of any biosynthetic product reflects not the specific dietary source of its precursors but rather the aggregate isotopic signature of all substrates entering the relevant pathways, weighted by their fluxes through those pathways [18].

Consider plasma alanine, the best-validated CIR [39]. Recent pooled analysis across 4 studies (*n* = 346) demonstrated correlations of *r* = 0.54 (95% CI: 0.46, 0.61) with added sugar intake and *r* = 0.63 (95% CI: 0.56, 0.69) with SSB intake across diverse populations [8]. A 2-wk controlled feeding study in 145 postmenopausal women with relatively low added sugar intake (median 48 g/d) found that CIR-alanine was associated with added sugar intake (*ρ* = 0.32, *P* < 0.0001), and when combined with CIR of glycine and isoleucine plus participant characteristics (body weight, physical activity, and smoking), achieved *R*^2^ = 0.37 [28]. Critically, this multivariate biomarker model was not associated with animal protein or red meat intake, suggesting improved specificity over whole-serum CIR approaches that conflate sugar and meat signals.

These associations persist across populations with different dietary patterns, though study-specific slopes vary substantially (0.34–1.75 for added sugar, 0.35–1.11 for SSB), indicating population-specific calibration may be necessary [8]. A 15-d–controlled feeding study further demonstrated that breath CIR responds to both added sugar and animal protein intake over multiple time scales, with strong correlations (*r* = 0.80) between midday and evening measurements but negligible association (*r* = 0.05, *P* = 0.60) between fasting breath CIR and habitual added sugar intake [9].

Alanine synthesis occurs via transamination of pyruvate, but the pyruvate pool receives carbon from multiple sources: glycolysis of dietary carbohydrates, glycolysis of glucose released from glycogen stores, gluconeogenesis from amino acids (particularly after protein-rich meals or during fasting), gluconeogenesis from lactate (particularly after exercise), and gluconeogenesis from glycerol (during fat mobilization) [40]. The relative contributions of these sources depend on feeding compared with fasting state, exercise status, insulin sensitivity, protein intake, and hepatic glycogen status [[41], [42], [43], [44], [45]]. Multiple metabolic factors influence stable isotope fractionation independent of dietary intake, including insulin resistance, altered glucose metabolism, body composition, inflammatory responses, metabolite pool sizes, metabolic branching events, and tissue-specific metabolic fluxes [46,47]. Two individuals consuming identical amounts of HFCS will exhibit different alanine isotopic values if one is insulin-resistant with elevated hepatic gluconeogenesis (contributing C3 carbon from amino acids to the pyruvate pool) and the other is insulin-sensitive, or if one exercises regularly (increasing lactate-derived pyruvate) and the other is sedentary. This variation is systematic rather than random noise, and importantly, metabolic phenotype correlates with the health outcomes that nutritional epidemiology seeks to understand, creating potential confounding by intermediate variables [48].

Breath CO_2_ derives from oxidation of acetyl-CoA in the citric acid cycle, but acetyl-CoA is produced from pyruvate via pyruvate dehydrogenase, from fatty acids via *β*-oxidation, and from ketogenic amino acids [[38], [39], [40]]. The isotopic composition of breath CO_2_ at any moment reflects the mixture of substrates being oxidized across multiple tissues, determined by nutritional state and metabolic demand rather than recent added sugar intake alone. During exercise, for example, fatty acid oxidation increases substantially, shifting breath CO_2_ toward the isotopic composition of body fat stores, which reflects long-term diet rather than recent intake [49].

Red blood cell proteins synthesized during erythropoiesis incorporate amino acids whose carbon skeletons derive from the complex mixture of dietary and metabolic sources active during the 120-d lifespan of the cells [50]. Hair and nail keratin similarly reflect aggregate metabolism over their growth period of weeks to months, not specific dietary added sugar intake during any particular time window [51,52]. These longer integration periods offer certain advantages for epidemiologic studies by reducing measurement error from day-to-day dietary variation, minimizing the influence of acute metabolic states (such as fasting, exercise, or glycemic control at a single time point), and better representing habitual dietary intake patterns. However, these advantages come with tradeoffs: reduced ability to detect recent dietary changes, inability to assess temporal patterns or dietary transitions, and greater potential for confounding by changes in food supply, agricultural practices, or geographic mobility during the extended integration period.

The key question is whether metabolic mixing introduces so much noise that dietary signal is lost, or whether dietary signal remains dominant despite metabolic complexity. The validation studies showing correlations of *r* = 0.59 between CIR of alanine and self-reported added sugar intake suggest that dietary signal is detectable in the populations studied [39]. However, we must ask: what proportion of biomarker variance reflects true added sugar intake compared with other sources of variation? When multiple sources of measurement error combine—including biological variation in isotope fractionation, analytical measurement error, and confounding by nontarget C4 sources—the cumulative uncertainty in biomarker-based intake estimates can be substantial. Even with moderate correlations between biomarker and intake in controlled settings (e.g., *r* = 0.70, *R*^2^ = 0.49), the unexplained variance (51% in this example) represents the combined influence of these error sources, which limits the precision and accuracy achievable in free-living populations. For most research applications, this degree of unexplained variation substantially limits utility [12].

Moreover, the *r* = 0.60 to 0.74 correlations are with self-reported added sugar intake, not true intake. If self-report has validity of *r* = 0.50 relative to true intake (optimistic based on validation studies using recovery biomarkers), then the correlation between CIR biomarkers and true intake may be substantially lower than reported validation coefficients suggest. The observed biomarker–self-report correlations might partially reflect shared errors or confounders rather than both methods validly measuring true intake.

Recent methodological frameworks for intake biomarker validation propose that a cross-validated *R*^2^ of ≥0.36 (correlation ≥0.60) between biomarker and feeding study intake represents adequate validity for nutritional epidemiology applications [53]. This criterion acknowledges that perfect biomarkers are unattainable and that even biomarkers explaining 36% of intake variance can provide value. However, the appropriateness of this threshold depends critically on the research application. For hypothesis generation and exploratory research, *R*^2^ = 0.36 may be adequate. For population surveillance requiring unbiased group-level estimates, or for etiologic research where substantial measurement error causes severe attenuation of disease associations, higher validity thresholds may be necessary. Moreover, this framework was developed primarily for nutrients where validation can be conducted without extensive dietary adjustment, whereas CIR added sugar biomarkers require adjustment for multiple confounding C4 sources, raising questions about whether the criterion applies when the biomarker–intake relationship is heavily dependent on covariate adjustment.

The Yun et al. [28] study provides a concrete example of how the *R*^2^ = 0.36 threshold performs in practice. In a controlled feeding study with low added sugar intake (median 48 g/d), the multivariate amino acid CIR biomarker achieved *R*^2^ = 0.37, just meeting the criterion. However, this required: *1*) measurement of 3 different amino acid CIRs using specialized gas chromatography–combustion IRMS (GC–CIRMS) rather than simpler whole-serum analysis, *2*) inclusion of 3 participant characteristics (body weight, physical activity, smoking), and *3*) a study population with minimal whole corn consumption. Whether *R*^2^ = 0.37 represents “adequate validity” depends critically on the research application and whether the added analytical complexity is justified by the marginal improvement over simpler approaches or well-designed self-report methods.

### The impossibility of adequate correction

#### Adjusting for mis-measured confounders

The standard response to specificity concerns is that statistical adjustment for measured confounders can isolate the added sugar signal. Validation studies routinely adjust for total corn intake, animal product consumption, and other C4 sources, reporting that added sugar remains significantly associated with CIR biomarkers after adjustment [39,54,55]. However, this approach faces serious methodological problems.

Adjustment requires accurate measurement of confounding variables, but these variables are assessed through the same self-report methods whose inadequacy motivated biomarker development in the first place. If self-reported added sugar intake has attenuation factors of 0.2 to 0.5, meaning 50% to 80% loss of association strength due to measurement error [2], we must accept comparable measurement error in self-reported corn intake, animal product intake, and other dietary components. Adjusting for mis-measured confounders produces unpredictable bias that can increase or decrease apparent associations depending on the correlation structure of measurement errors [56,57].

Consider a simplified scenario: true added sugar intake (Y) and true corn intake (C) both affect the biomarker (B), and we observe error-prone measures of both dietary exposures. If errors in reported sugar and reported corn are positively correlated, which is plausible because they arise from the same cognitive processes and social desirability biases, then adjusting for reported corn intake can actually increase bias in the apparent sugar–biomarker association rather than reducing it. The direction and magnitude of bias depend on error correlation structures that are difficult to estimate empirically [56].

Moreover, the residual correlation between CIR biomarkers and added sugar after adjustment for confounders demonstrates only that added sugars and other C4 sources are imperfectly correlated in the validation population, not that the biomarker specifically measures added sugars independent of those sources. If individuals who consume high levels of HFCS happen to consume low levels of corn tortillas due to different ethnic dietary patterns, added sugar will explain biomarker variance after adjusting for tortillas. This reflects demographic patterns of food choice, not biomarker specificity. In a different population where HFCS consumers also consume substantial corn foods (e.g., Mexican-American populations), the biomarker might show no residual association with added sugar after adjustment.

#### The growing list of required adjustments

The number and complexity of required adjustments have grown as researchers have recognized additional sources of confounding [3,12,58]. Future research will undoubtedly identify additional confounders, including corn oil consumption, diverse corn-derived food additives, metabolic phenotype indicators, and possibly genetic variants affecting carbon routing through different pathways.

Information theory provides a formal answer to when adjustment renders a biomarker superfluous: if the biomarker is conditionally independent of true intake given measured covariates, then it provides no information about intake beyond that already contained in the covariates [57,59]. Validation studies requiring extensive covariate adjustment to demonstrate biomarker–intake associations are approaching this condition of conditional independence [60]. At this point, the “biomarker” becomes a complex regression model where the isotope measurement provides marginal information beyond dietary assessment and phenotyping. The biomarker has lost its fundamental value proposition, even if technical validation metrics appear acceptable.

#### Logical circularity

A deeper problem is logical circularity: we cannot validate that CIR biomarkers specifically measure added sugars without already possessing accurate measures of all confounding C4 sources. The biomarker was motivated by inadequacies of self-report [1], yet its validation and proper use require accurate self-report of dietary components that self-report measures poorly. This circularity is not merely inconvenient but rather suggests a fundamental logical problem with the biomarker approach for this application. Contrast this with recovery biomarkers like doubly labeled water for energy expenditure, where physical laws constrain total energy metabolism and the biomarker measures total energy independently of any dietary assessment [61]. CIR added sugar biomarkers have no such independence; their interpretation as measures of added sugar intake is inherently conditional on knowledge of other dietary components [12,13].

### Validity requirements for different research applications

The level of validity required from CIR biomarkers depends on how they are used in research, and their suitability varies considerably across applications.

For *population surveillance and monitoring of trends*, such as tracking changes in added sugar consumption over time in population nutrition surveys, the main goal is to obtain unbiased estimates of group means and trends rather than precise rankings of individuals [62]. CIR biomarkers struggle to meet this need. Temporal instability is a major concern because the C4/C3 sugar ratio has shifted over time due to changes in sweetener sources, like the replacement of cane and beet sugar with HFCS, and continues to fluctuate with market conditions. A downward trend in CIR values might suggest lower sugar consumption, yet it could just as easily reflect changes in sugar sourcing or substitution with C3-based sweeteners like honey and fruit juice concentrates. Disentangling these influences requires detailed food supply data. Cohort effects further complicate interpretation, because different generations have varying dietary patterns and sources of animal products, leading to confounding between age, period, and cohort differences [63]. Population heterogeneity adds another layer of difficulty; as food systems globalize and diets diversify, the assumption of minimal whole corn consumption becomes less reliable. Surveillance efforts would either need to restrict analysis to specific subgroups or collect detailed dietary data for adjustment, both of which undermine the intended simplicity of using a biomarker. Overall, CIR biomarkers are poorly suited for surveillance due to their temporal instability and the need for extensive auxiliary information to achieve unbiased estimates.

For *etiologic research investigating sugar–disease associations*, validity requirements depend on the true effect size, confounding, and desired precision. If the true hazard ratio is around 1.5 per daily serving increase in added sugars and confounding is limited, a biomarker with a correlation of 0.50 with true intake would reduce the observed hazard ratio to ∼1.22, leading to a significant loss of statistical power. CIR biomarkers are prone to several sources of bias in this setting. Metabolic phenotypes that influence CIR, such as insulin resistance or gluconeogenesis rate, are also linked to disease risk, introducing confounding that is difficult to correct analytically. Dietary pattern confounding is another problem because CIR values reflect consumption of C4-based processed foods more broadly, not added sugars specifically [12,13]. Even assuming reasonable validation correlations with self-reported intake, the effective correlation with true intake may be substantially lower, leading to attenuation of observed associations toward the null [64]. The degree of attenuation depends on the magnitude of measurement error in the exposure variable. CIR biomarkers may still be useful when studying large effects (hazard ratios >2.0), provided that metabolic confounding is addressed carefully, but they are likely inadequate for detecting moderate associations. The variability in reported *R*^2^ values across studies (0.36–0.91) [6] illustrates that biomarker validity is highly context-dependent. The highest *R*^2^ values (0.87–0.91) come from controlled feeding studies with uniform dietary exposures and minimal confounding [65], whereas studies in free-living populations with diverse dietary patterns show more modest correlations. This pattern suggests that the biomarker performs well in capturing C4 dietary carbon exposure under controlled conditions but faces greater challenges when multiple C4 sources vary independently in real-world settings. The null association between fasting breath CIR and habitual added sugar intake in the O’Brien et al. [9] feeding study, despite strong associations in postprandial measurements, further demonstrates that timing, metabolic state, and specimen type substantially influence biomarker performance.

In *dietary intervention trials*, where the goal is to detect within-person changes after a sugar-reduction intervention, randomization removes between-person confounding [66], shifting the focus to whether CIR biomarkers respond proportionally to changes in intake. Reliability and responsiveness are key challenges. If biological variation exceeds dietary signal, test–retest reliability may be poor, limiting the ability to detect intervention effects. Compliance variability also matters. Participants who cut added sugars but increase whole corn or corn-fed animal products might show muted biomarker changes, misleadingly suggesting weak adherence. Moreover, different biomarker compartments (plasma glucose, alanine, red blood cell proteins, hair) reflect intake over varying time windows [67], so matching the biomarker’s integration period to the intervention duration is essential. CIR biomarkers may perform best in trials that specifically target C4 sugars (e.g., SSBs containing HFCS), explicitly discourage substitution with other C4 foods, and empirically verify within-person reliability in pilot work. Still, complementary self-report or objective intake measures are necessary to interpret observed biomarker changes with confidence.

### Alternative explanations for observed correlations

If CIR biomarkers do not validly measure added sugar intake, why do validation studies consistently report moderate correlations with self-reported added sugar intake? Several mechanisms can account for these observations without requiring biomarkers validly and specifically measure added sugars.

#### Dietary pattern coherence

Individuals who consume high levels of added sugars tend to consume generally westernized, processed diets characterized by: processed snack foods often containing corn-based ingredients, fast food cooked in corn oil and containing corn-derived starches, sweetened beverages with HFCS, convenience foods with multiple corn-derived additives, and industrially produced meat from corn-fed animals [68,69]. The correlation between CIR biomarkers and reported sugar intake thus partly reflects coherent dietary patterns rather than specific measurement of sugars. Both the CIR biomarker and reported added sugar intake are elevated in individuals consuming highly processed diets rich in diverse C4-derived ingredients. The amino acid CIR approach attempts to address this by using the distinct metabolic fates of nonessential compared with essential amino acids to differentiate sugar from meat signals [28], but this improvement in biochemical specificity comes at the cost of increased analytical complexity and continued dependence on participant characteristics for adequate predictive validity.

This mechanism would produce positive correlations in validation studies without the biomarker specifically measuring added sugars. Importantly, this explanation predicts that correlations would be stronger in populations with more coherent processed-food dietary patterns and weaker in populations where sugar consumption and other C4 sources are less correlated.

#### Socioeconomic structuring

Both CIR biomarkers and added sugar intake are structured by socioeconomic status, ethnicity, geographic location, and food environment [3,70]. Lower-income individuals have greater access to inexpensive processed foods high in both HFCS and other corn-derived ingredients, less access to whole foods and premium products using C3 sugars, and different animal product sourcing (conventional grain-fed compared with premium grass-fed) [71,72]. Higher-income individuals show opposite patterns. These shared socioeconomic determinants create a correlation between CIR biomarkers and reported intake that does not necessarily reflect causal measurement validity. This confounding structure is particularly problematic because socioeconomic status also affects the metabolic outcomes that sugar–disease research seeks to understand [73], potentially creating spurious associations when CIR biomarkers are used in etiologic studies. It should be noted that any metabolic or health effects associated with C4 intake would be attributable to the specific foods consumed (corn-derived products, corn-fed animal products, cane sugar) rather than to isotopic composition per se, because no evidence exists for differential biological effects of ^12^C compared with ^13^C isotopomers given the negligible mass difference relative to molecular weight.

#### Metabolic confounding

Metabolic factors that affect appetite and eating behavior also affect carbon metabolism. Insulin resistance increases subjective cravings for sweet foods while also altering hepatic gluconeogenesis and amino acid metabolism [74,75]. This creates a correlation between reported sugar intake and CIR biomarker values that reflects shared metabolic causes rather than CIR biomarkers measuring intake. Leptin resistance, genetic variants affecting sweet taste perception, and other metabolic factors similarly create potential for spurious correlation [76,77].

Validation studies that adjust for BMI and sometimes insulin resistance partially addresses this confounding, but residual confounding almost certainly remains. The correlation between CIR biomarkers and self-report after adjustment for measured metabolic factors may partially reflect unmeasured metabolic heterogeneity [78].

#### Empirical evidence needed

These alternative explanations are plausible but largely speculative without direct empirical evidence. Distinguishing between true biomarker validity and these alternative mechanisms would require:

1. Validation studies in populations where added sugars and confounding C4 sources are uncorrelated (or negatively correlated), testing whether biomarker–sugar correlations persist.
2. Genetic epidemiology studies using Mendelian randomization to separate metabolic from dietary effects on CIR biomarkers.
3. Controlled feeding studies with manipulation of added sugars while holding all other C4 sources constant over a sufficient duration to reach isotopic steady state.
4. Animal model studies to elucidate isotope incorporation and fractionation mechanisms under controlled conditions, recognizing that translation to humans requires validation because of species differences in metabolism and inability to model complex human food systems.
5. Preregistration of validation study protocols to prevent analytic flexibility from inflating reported correlations.

Such studies would help quantify how much of observed validation correlations reflect true biomarker validity compared with confounding and bias. In the absence of this evidence, the moderate correlations in published validation studies remain ambiguous and should not be uncritically interpreted as demonstrating biomarker utility.

### When might CIR added sugar biomarkers provide value?

Although CIR biomarkers have substantial limitations for most common research uses, there are specific circumstances where they might still offer value.

#### Within-person change in intervention studies

In dietary intervention trials where participants act as their own controls, such as crossover designs, between-person confounding by corn intake and animal product consumption is less problematic if those dietary components remain stable while added sugar intake changes. For example, if an intervention successfully reduces added sugar intake by ∼50 g/d while corn tortilla and meat consumption remain constant, the CIR biomarker should capture this change, even though its absolute value reflects multiple dietary sources. The amino acid CIR approach, which uses CIR of alanine combined with CIR of essential amino acids and participant characteristics, may offer improved specificity in this context by distinguishing sugar-derived signals from meat-derived signals [28]. However, this requires more complex analytical methods (GC–CIRMS for individual amino acids rather than whole-serum analysis) and multivariate modeling, increasing both cost and methodological complexity relative to simpler whole-serum approaches.

This type of application depends on several conditions. The intervention must specifically target added sugars without inadvertently altering other C4 sources. Within-person reliability must be high, meaning metabolic variation contributes minimally to biomarker variability relative to dietary changes. The biomarker compartment chosen must integrate intake over the same time frame as the intervention period, and compliance heterogeneity should be assessed to interpret null results correctly. Pilot studies establishing within-person test–retest reliability under stable dietary conditions are essential before using CIR biomarkers as primary outcomes in trials.

#### Populations with particularly poor self-report

Certain populations have such severe limitations in dietary self-report that even imperfect biomarkers may provide incremental value. Young children cannot accurately report their own food intake [79], and parent proxies often lack information about foods eaten outside the home [80]. Elderly individuals with cognitive impairment and adults with low literacy face similar barriers to reliable self-reporting [[81], [82], [83], [84], [85]]. In these populations, the alternative to CIR biomarkers may be essentially no valid dietary data at all. Even biomarkers explaining 30% to 40% of the variance in true intake could outperform self-report methods that explain <20%. However, researchers must consider whether confounding from corn consumption or corn-fed animal products is equally problematic in these groups. If these confounders vary substantially, interpretation of CIR biomarkers remains uncertain.

#### Triangulation and measurement error correction

Biomarkers with modest validity can still contribute when combined with self-report in measurement error correction frameworks [86]. Self-report and biomarkers differ in their error structures [82]. Self-report is prone to recall bias, social desirability bias, and portion-size errors [87], whereas biomarkers suffer from metabolic heterogeneity and dietary confounding [67]. If these errors are uncorrelated, combining the 2 can improve estimation accuracy. Regression calibration and similar methods use biomarkers to adjust self-reported intakes for measurement error [88]. These approaches assume that the biomarker is unbiased or that any bias can be characterized. Given the specificity issues surrounding CIR added sugar biomarkers, this assumption may not hold, but simulation studies could help quantify the effects of different bias patterns. Applying these methods requires advanced statistical modeling and transparent communication about underlying assumptions, with researchers clearly stating how both self-report and biomarker validity influence the final combined estimate.

#### Objective outcomes in trials where blinding is critical

In trials where participant awareness of treatment assignments could bias self-reported outcomes, objective biomarkers can help mitigate that bias. Even if CIR added sugar biomarkers are imperfect, a biomarker with a correlation of 0.50 to true intake that is unaffected by participant awareness may be preferable to self-report data with slightly higher validity but substantial bias from unblinding. This application only works if the intervention does not alter metabolism in ways that affect CIR biomarker interpretation. For example, if a sugar-reduction intervention also increases physical activity, which influences carbon metabolism, the resulting biomarker change may reflect more than just dietary change. In such cases, measuring and adjusting for physical activity becomes necessary, reintroducing some dependence on self-reported data.

The above potential niche uses of CIR biomarkers share several features. They arise in contexts where alternative methods are limited, require careful pilot testing to establish feasibility and reliability, depend on assumptions that must be validated empirically, and still rely on some dietary data for interpretation. Their value lies in providing incremental improvements rather than transformative advances. Researchers should explicitly evaluate whether their specific study conditions justify the use of CIR biomarkers rather than assuming general validity. Demonstrating utility should rest on evidence from the intended application rather than extrapolation from unrelated validation studies.

### Comparison to successful isotope applications

The limitations of CIR biomarkers for added sugars should not be seen as a critique of stable isotope methods overall. Carbon and nitrogen isotopes have proven effective in dietary studies where isotopic signals align with meaningful nutritional categories [3]. For example, combined δ^13^C and δ^15^N analysis clearly distinguishes marine from terrestrial protein intake because of distinct isotopic baselines and minimal confounding, directly reflecting differences like omega-3 consumption [3,11]. Similarly, δ^15^N values in hair and blood correlate with animal compared with plant protein intake, with trophic level enrichment providing a reliable signal despite minor confounders [10,11,51].

These successes stem from a strong match between isotopic variation and the dietary exposures of interest. In contrast, CIR biomarkers for added sugars struggle because δ^13^C differentiates C3 and C4 plants, not added compared with natural sugars [12,13]. The C4 signal is confounded by multiple sources, such as corn, corn-fed animals, and various corn-derived ingredients, each with different nutritional implications. Moreover, carbohydrate-based biomarkers are less stable than protein-based ones, and evolving public health definitions (e.g., from “added” to “free” sugars) further weaken the alignment between isotopic signals and target exposures [12,13]. The issue lies not in isotope science itself, but in its application to a poorly matched nutritional question.

### Resource allocation and alternative approaches

#### Costs and opportunity costs

Developing and validating CIR biomarkers for added sugars have required substantial resources. IRMS typically costs $15 per sample for analysis [89], but when factoring in preparation and overhead, total costs often reach $50 to $100 per participant. By comparison, a single 24-h dietary recall costs $15 to $30, and a food frequency questionnaire (FFQ) costs $10 to $20. Both methods offer comprehensive data on the entire diet, making the cost attributable to added sugars negligible.

Because validation studies require dietary data for confounding adjustment, combining CIR biomarkers with self-report can raise total costs to $75 to $125 per participant. Although smaller sample sizes can partly offset costs, because statistical power scales with the square of measurement validity, the per-sample cost of CIR biomarkers remains high. For example, if CIR validity is 0.60 and self-report validity is 0.40, a biomarker-based study could achieve equivalent power with 56% of the sample size, but at 2 to 3 times the total cost. These differences become even less favorable when considering that the best-practice self-report involves multiple recalls, the CIR validity of 0.60 may be optimistic, and dietary data are still needed for adjustment. Moreover, self-report methods provide richer information on dietary patterns than biomarkers. Given these tradeoffs, researchers must weigh whether the incremental validity gain from CIR biomarkers justifies the substantially higher financial investment.

#### Improved self-report methods

Technological innovations have begun to enhance self-report methods. Image-based assessment using smartphone photography with artificial intelligence–driven food recognition can improve portion size and food identification accuracy, with current systems achieving ≤96% accuracy [[90], [91], [92]]. Passive sensing through wearable devices can detect eating episodes and prompt participants to capture brief records, reducing recall bias [93]. Ecological momentary assessment using short smartphone surveys throughout the day minimizes recall delay and captures contextual information [94]. Although these technologies face challenges, such as compliance, access disparities, and incomplete data capture, they address core weaknesses of traditional self-report methods and maintain specificity for nutrients, including added sugars. Investing in these tools may yield greater long-term benefits than continuing CIR added sugar biomarker development.

#### Food purchase data

Data from retail loyalty programs and credit cards provide large-scale, objective records of food acquisition. These data include ingredient-level nutrient information, impose no participant burden, and allow near real-time tracking of dietary trends [95]. Although they require calibration for household-level compared with individual intake and miss food wastes and foods consumed away from home, they are powerful tools for population surveillance, trend monitoring, and policy evaluation [95]. They complement rather than replace individual-level dietary assessment but offer advantages that isotope biomarkers cannot match.

#### Metabolomic markers

Metabolomic approaches aim to measure the biochemical footprint of added sugar consumption by detecting sugar molecules or their metabolic byproducts in biological samples [7]. Candidate biomarkers include serum or urinary fructose and sucrose, which are compounds directly linked to added sugar, as well as downstream metabolites such as mannose, uric acid, and advanced glycation end-products [7]. Composite metabolomic profiles may also enhance specificity [96].

Although these markers offer a more direct link to sugar metabolism than isotope-based methods, they are not fully specific to added sugars [12]. Individual sugar types like fructose and sucrose can also originate from naturally occurring sources such as fruits and vegetables, making it difficult to distinguish added from natural sugars based solely on their presence [97]. Additional limitations include rapid clearance (requiring precise sample timing) and poor reproducibility over time [7]. Nonetheless, metabolomics holds greater theoretical potential for specificity than CIR added sugar biomarkers, which rely on plant photosynthetic pathways unrelated to sugar type. With improved understanding of sugar metabolism, identification of more stable and context-specific markers, and multimetabolite strategies, metabolomics may offer a more promising path forward [7,96], though it is unlikely to achieve perfect specificity for added sugars.

All of the above alternatives to CIR added sugar biomarkers come with drawbacks (Table 2). The field lacks a clearly superior solution for dietary assessment generally or added sugar assessment specifically. Given this reality, some researchers may reasonably conclude that CIR added sugar biomarkers, despite their limitations, provide value by offering a measurement with different error characteristics than alternatives [3,6,9]. This position has merit but requires explicit acknowledgment of biomarker limitations and careful consideration of whether the specific research context favors biomarker use. The argument here is not that alternatives are perfect, but rather that CIR biomarkers’ fundamental specificity problems make them unlikely candidates for major resource investment when alternatives have challenges that are potentially more tractable [13].

**TABLE 2:** Comparison of dietary assessment methods

Method	What it actually measures	Specificity for added sugars	Cost per assessment	Major limitations	Can limitations be overcome?	Verdict for added sugar assessment
24-h dietary recall	Self-reported foods consumed in past 24 h	High. Can identify specific sources (SSBs, candy, baked goods)	Low (self-administered) Moderate (interviewer administered)	Social desirability bias; memory errors; portion-size estimation	Partially. Multiple recalls, technology-assisted methods, biomarker calibration reduce error	Acceptable with improvements. Imperfect but provides detail biomarkers cannot match
Food frequency questionnaire	Self-reported usual intake over months	High. Asks about specific food sources of added sugar	Low (self-administered) Moderate (interviewer administered)	Conceptualization of “usual” intake; poor portion-size estimation; limited food lists	Partially. Updated food lists, portion-size aids, validation studies improve performance	Acceptable for ranking. Best for relative intake in large studies despite known errors
Food photography/smartphone apps	Researcher-observed actual foods via images	Very high. See exact foods, brands, preparation	High initial cost Low collection/analysis costs	Requires compliance; does not capture all eating occasions; image quality varies	Yes. AI recognition improving rapidly; automated portion estimation	Promising. Addresses key self-report limitations while maintaining specificity
Food purchase data	Actual foods acquired (loyalty cards, credit cards)	Very high. Exact products with complete ingredient data	Low (data access cost only)	Does not capture food waste; eating outside home; household vs. individual	Partially. Combine with recalls to calibrate; focus on population-level estimates	Promising for population surveillance. Objective with superior specificity to biomarkers
CIR biomarkers (δ^13^C)	Total C4 dietary carbon from all sources after metabolic mixing	Low. Confounded by corn foods, corn-fed meat, beet sugar; source identity lost in metabolism	High	Lack of specificity; metabolic redistribution; population-specific validity; validation circularity	No. Limitations are fundamental, not technical	Not acceptable for most uses. Expensive, lacks specificity, requires extensive self-report for “correction”
Metabolomic markers	Specific metabolic products of sugar metabolism	Moderate. Reflects actual sugar metabolism, not just C4 sources, but does not distinguish between added vs. natural sources	High	Rapid clearance; requires precise timing; nonspecific elevation by multiple sources	Maybe. Discovering more specific markers or composite metabolic profile; better understanding of metabolism	Potentially promising. Has path to true specificity unlike CIR
Urinary sucrose/fructose	Unmetabolized sugars excreted	Moderate. Actual sugar molecules, but does not distinguish between added vs. natural sources	Moderate	Low recovery (most sugar metabolized); high variability; requires multiple samples	Maybe. Better understanding of excretion patterns; multiple timepoints	Limited utility but specific for individual sugar types. Mixed signal but measures actual target

Abbreviations: AI, artificial intelligence; CIR, carbon isotope ratios; SSBs, sugar-sweetened beverages.

### Lessons for biomarker development

The CIR biomarker experience highlights key principles for future biomarker development. Moderate correlations with self-reported intake are insufficient evidence of validity. They may reflect shared confounders, dietary pattern coherence, or analytic bias. True validation requires controlled feeding studies that isolate intake effects, demonstrate dose–response relationships, predict relevant outcomes, and rule out alternative explanations [98].

Initial optimism that δ^13^C could reflect added sugar intake has not held up empirically. Developers must rigorously identify all dietary and metabolic contributors to biomarker variation and assess whether these can be accounted for without undermining the marker’s simplicity. Specificity must be demonstrated empirically, and within each population studied, because the performance of a biomarker is population dependent due to differences in food systems, agricultural practices, dietary patterns, and metabolic phenotypes [67]. A biomarker validated in one group may fail in another [67]. Population-specific validation is essential, not optional, before application.

This presents a practical challenge: comprehensive validation across diverse populations demands substantial resources, which may outweigh the benefits. Researchers must weigh the cost of validation against the incremental value biomarkers offer over alternatives. Even moderately valid biomarkers may not be cost-effective. Investment decisions should be guided by cost-effectiveness analyses that consider direct, indirect, and opportunity costs, as well as the informational value added.

Validity requirements also vary by application. Surveillance needs unbiased group-level estimates; etiologic research requires sufficient validity to detect true associations; trials need reliable within-person responsiveness; and clinical use demands accurate individual-level estimates. Biomarker adequacy must be judged relative to its intended purpose.

Although CIR biomarkers fall short for added sugar research, their development has advanced understanding of isotopic variation, human carbon metabolism, and validation methodology. Even unsuccessful approaches yield valuable insights that inform future innovation in dietary measurement.

In conclusion, CIR biomarkers for added sugar intake face fundamental limitations that compromise their validity in most research settings. Recent systematic reviews and controlled feeding studies have established that CIR-alanine represents the most promising biomarker currently available, with moderate correlations to added sugar intake across diverse populations. However, these biomarkers measure total C4 dietary carbon, not added sugars specifically, and are confounded by other C4 sources, such as corn-based foods and corn-fed animal products, whose contributions vary across populations and food systems. The distinction between “most promising available biomarker” and “sufficiently valid for broad application” is critical. Although niche applications may justify their use, this requires careful design, pilot validation, and transparent communication of assumptions.

The broader lesson is clear: biomarker development must prioritize specificity, empirical validation across diverse populations, and cost-effectiveness. Only biomarkers that deliver information truly independent of, or substantially better than, existing methods justify the resources required. CIR biomarkers for added sugar, despite moderate correlations, generally fail to meet these criteria. Even after considerable investment, they remain unlikely to do so. Future efforts may be better directed toward metabolomic approaches, improved self-report tools, and integrated methods that offer clearer paths to valid dietary assessment.

## Author contributions

The sole author was responsible for all aspects of this manuscript.

## Declaration of Generative AI and AI-Assisted Technologies in the Writing Process

During the preparation of this work, the author used Claude 4.5 sonnet (https://claude.ai) in Oct 2025 to improve the grammar and clarity of the text he drafted. After using this tool/service, the corresponding author reviewed and edited the content as needed and takes full responsibility for the content of the publication.

## Funding

The author reported no funding received for this study.

## Conflict of interest

The author reports no conflicts of interest.
